# Practice and factors associated with neonatal sunlight exposure among mothers in Jigjiga City, Somali Regional State, Eastern Ethiopia

**DOI:** 10.1186/s12887-025-05776-5

**Published:** 2025-05-31

**Authors:** Amal Adem Abdulahi, Mohamed Omar Osman, Yahye Mohamed Ibrahim, Ramadan Budul Yusuf, Ahmed Mohammed Ibrahim, Seid Muhumed Abdilaahi, Yusuf Hamud File, Nour Ahmed Korane, Ahmed Adem Abdullahi

**Affiliations:** 1 Departments of Pediatrics and Child Health Nursing, Institute of Health Science, Jigjiga University, Jigjiga, Ethiopia; 2 Departments of Public Health, Institute of Health Science, Jigjiga University, Jigjiga, Ethiopia; 3 Department of Medicine, Institute of Health Science, Jigjiga University, Jigjiga, Ethiopia

**Keywords:** Practice, Sunlight exposure, Neonates, Factors associated, Jigjiga, Eastern Ethiopia

## Abstract

**Background:**

Sunlight is a vital natural resource for sustaining life on Earth. Sunlight exposure, especially ultraviolet B, is necessary for synthesizing vitamin D in the skin. Early life is the most prevalent time for vitamin D deficiency, and almost half of mothers in Ethiopia are estimated to have a low level of good practice of sunlight exposure, which remains a major contributing factor to vitamin D deficiency in Ethiopian children.

**Objectives:**

To assess the practices and factors associated with neonatal sunlight exposure among mothers in Jigjiga City, Somali Regional State, Eastern Ethiopia, 2024.

**Methods and materials:**

A community-based cross-sectional study was conducted among 417 mothers in Jigjiga city from April 16 to 30, 2024. The study participants were selected using a systematic random sampling technique. The data were collected using electronic data collection software (Kobo) and exported to SPSS software version 20 for analysis. Bivariable and multivariable binary logistic regression was performed. Finally, the adjusted odds ratio with a 95% confidence level was computed, and a p-value < 0.05 was used to declare statistical significance.

**Results:**

Among the mothers interviewed, 29.4% with 95% CI (22.2–38.1) had good practice in exposing their neonates to sunlight. In multivariable analysis, mothers who were married (AOR = 0.17; 95% CI: 0.03–0.92), who delivered through caesarian section (AOR = 0.05; 95% CI: 0.01–0.54), who had poor knowledge of sunlight exposure (AOR = 0.31; 95% CI: 0.1–0.96) and who had no fear of sunlight exposure (AOR = 3.06; 95% CI: 1.02–9.17) were significantly associated with good practices of neonatal sunlight exposure.

**Conclusions:**

This study revealed that 29.4% of mothers had good neonatal sunlight exposure practice which is low compared to other studies. The mothers’ marital status, mode of delivery, mother’s knowledge, and fear of sunlight exposure were significantly associated with the practice level of the mothers.

## Introduction

Sunlight is a vital natural resource that is essential for sustaining life on Earth; it provides both warmth and light, without which human survival is impossible [1]. It has long been used for therapeutic purposes, also known as heliotherapy, dating back to ancient Greece and Rome [2]. Sunlight was discovered to have a bactericidal effect and was able to treat rickets in the second half of the 19th century [3, 4].

Ultraviolet Radiation (UVR), especially Ultraviolet B radiation, is necessary for the synthesis of vitamin D in the skin [5]. The sunshine vitamin, vitamin D, is indispensable for fostering the growth and strength of healthy bones by keeping calcium and phosphate plasma concentrations within the normal physiological range and working with parathyroid hormone to maintain bone mineralization [6, 7].

Exposing the skin to sunlight produces approximately 80–90% of vitamin D in humans; in addition to being produced by the body when exposed to sunshine, vitamin D can also be found in eggs, fatty salmon, and fortified foods [8]. Vitamin D deficiencies (VDD) due to inadequate sunlight exposure can lead to conditions such as rickets, which are characterized by weakened and deformed bones, as identified by different studies globally [9].

Insufficient sun exposure has emerged as a significant public health concern, highlighting the need for a timely and essential shift in current sun avoidance guidelines to ensure adequate exposure, as even a small change can have a critical impact on overall health outcomes [10]. A global pandemic of VDD has resulted from the general lack of recognition regarding the importance of responsible sun exposure in fulfilling the vitamin D requirements of both adults and children [6].

In low- and middle-income countries, dietary foods that provide vitamin D and are the source of vitamin D are frequently not easily accessible [11]. Consequently, inadequate sunlight exposure in a nation without or with little vitamin D supplementation leads to VDD, which is linked to various health conditions such as rickets, skeletal demineralization, cardiovascular disease, cognitive decline, depression, adult-onset diabetes mellitus, osteoporosis, osteoarthritis, autoimmune diseases, growth retardation and skeletal abnormalities in children, and infantile eczema in young children [12]. However, it also remains one of the major contributing factors to VDD in Ethiopian children, as does an increase in infant mortality and morbidity [13, 14].

According to different studies conducted in Ethiopia, the level of good practice, ranging from 22 to 44.6%, with respect to infants’ sunlight exposure [15–20]. Various factors, such as the mother’s age, education level, family size, husband’s education level, marital status, occupation, fear of the evil eye, place of delivery, neonate’s age, Antenatal Care (ANC) visit and knowledge of mothers regarding sunlight exposure were significantly associated with the practice of mothers regarding the sunlight exposure of infants [15, 20–23].

Globally, recommendations to prevent VDD include adequate sunlight exposure, vitamin D supplementation, and food fortification [24]. Public health interventions such as infant vitamin D supplementation are also recommended for settings with high ricket prevalence; however, Ethiopia does not have such a program [11]. In Ethiopia, strategies to address VDD and rickets have focused on health education to encourage mothers to expose infants to sunlight and newborn sunning guidelines; however, these strategies have not been consistently implemented across the country [14, 25].

Although mothers play a crucial role in preventing rickets, few studies have been conducted on the practices and factors associated with mothers’ level of sunlight exposure, particularly in this study area. In addition to that scarcity of studies, mode of delivery was not investigated in previous studies. Therefore, this study aims to assess the practices and factors associated with neonatal sunlight exposure among mothers in Jigjiga city, Somali Regional State, Eastern Ethiopia, 2024.

## Methods and materials

### Study area and period

The study was conducted in Jigjiga city, the capital city of the Somali regional state, which is located approximately 629 km southeast of Addis Ababa, the capital city of Ethiopia, and 60 km west of the border with Somalia. According to the 2007 census conducted by the Central Statistical Agency of Ethiopia, Jigjiga has a total population of 277,560, of whom 149,292 are men and 128,268 are females [26]. The city administration has one specialized comprehensive hospital, two public hospitals, and three health centers. It also has 21 urban kebeles. The study was conducted from April 16 to 30, 2024.

### Study design

A community-based cross-sectional study was conducted.

### Population

#### Source population

All mothers who had neonates and lived in Jigjiga city.

#### Study population

All randomly selected mothers and their neonates who lived in Jigjiga city during the study period.

### Inclusion and exclusion criteria

#### Inclusion

All mothers who had neonates and had been living in Jigjiga city for at least 6 months.

#### Exclusion

Mothers who were unable to speak (severely ill).

Neonates being cared by caregiver.

### Sample size determination

For the first objective, the sample size was calculated using a single population proportion formula by considering the following assumption:

P: Prevalence of mothers’ practice regarding sunlight exposure from previous study: 44% [15].

d: Margin of error tolerated: 5% (0.05).

Z α/2 Confidence level at: 95% Confidence Interval (CI): (1.96)

n: Sample size.

$$n = \frac{{{{\left( {Z\alpha /2} \right)}^2}P\left( {1 - P} \right)}}{{{d^2}}}$$

$$n = \frac{{{{\left( {1.96} \right)}^2} \times \left( {0.44} \right)\left( {1 - 0.44} \right)}}{{{{\left( {0.05} \right)}^2}}} = 379$$

$$n = 379{\text{ }} + {\text{ }}37.9{\text{ }}\left( {10\% } \right)$$

Therefore, the final sample for this study is 417.

### Sampling technique and procedure

From the 21 urban kebeles of Jigjiga city, 6 kebeles (30%) were selected using simple random sampling through lottery method. The calculated sample size was then proportionally allocated to each selected kebele on the basis of the total list of households in the kebele. The study participants in each kebele were selected using systematic random sampling technique after the sampling interval (K = 4) was calculated. The first household was selected by lottery method from the first four households. If the selected household was not eligible, the next household was selected. If there were two neonates in one household, only the mother or caretaker of one neonate was selected by lottery method. A second visit was made if eligible respondents were not available at the time of the data collection.

### Data collection tools and techniques

Data were collected with face-to-face interview using pretested and semi structured questionnaires adapted from different studies [15, 20–23]. The questionnaire addresses the following questions: sociodemographic factors (mother’s age, infant’s age, marital status, mother’s educational status, mother’s occupation, family size, husband’s educational status), maternal related factors (ANC follow up, place of delivery, mode of delivery), mothers’ knowledge of sunlight exposure, which includes 7 questions, mothers’ practice of sunning their neonates, which includes 10 questions and behavioral factor (mother’s fear of evil eye, sickness and others).

### Data collection procedures

Data were collected from mothers who had neonates through face-to-face interview using a semi-structured questionnaires and house–to–house visits in the selected kebeles of Jigjiga city. Three data collectors and one supervisor were recruited, and one-day training was given to them about the objective, relevance of the study, informed consent, confidentiality of information, and technique of the interview.

### Study variables

#### Dependent variable

Practice of sunlight exposure – Good/ Poor.

#### Independent variables

Sociodemographic factors: mother’s age, infant’s age, marital status, mother’s educational status, mother’s occupation, family size, husband’s educational status.

Maternal-related factors: ANC follow-up, place of delivery, mode of delivery.

Mothers’ knowledge of sunlight exposure.

Behavioral factor: mother’s fear of evil eye, sickness and others.

### Data quality control

The data collection tool was pretested on 5% of the total sample size (*n* = 21) to assess its clarity, completeness, consistency and required time to carry out the interview. The pretest was conducted in Kebribeyah town, Fafan Zone to avoid information contamination. The data were checked for completeness on a daily basis by the supervisor and principal investigator, and finally, the data were cleaned before analysis.

### Operational definitions

Knowledge: Mothers’ theoretical understanding of neonatal sunlight exposure [20].

Good Knowledge: Mothers who responded to knowledge questions (such as information about sunlight exposure, the benefit of sunlight, the harm of sunlight and a good time of sunlight exposure) and who scored equal to or above the median value of 6.

Poor Knowledge: Mothers who responded to knowledge questions and scored below the median value of 6.

Practice: Mothers’ activity or behavioral experience in relation to the sunlight exposure of neonates [20].

Good Practice: those mothers who responded to all practice questions correctly and as follows (start at 2 weeks of age, outdoors, at least 3 times a week, minimum of 15–30 min, unclothed, morning at 8–10 AM and apply any lubricants after exposure).

Poor Practice: those mothers who responded to all practice questions other than those stated above.

### Data processing and analysis

The data were collected using electronic data collection software (Kobo) and then downloaded and exported to Excel, and SPSS software version 20 was used for statistical analysis. Descriptive statistics, including frequency distributions, proportions, and measures of central tendency, were used to summarize the data. Binary logistic regression was used to assess the associations between independent variables and mothers practice towards neonatal sunlight exposure. Bivariable binary logistic regression was used and those variables with a p-value < 0.25 were selected as candidates for multivariable binary logistic regression analysis. Multicollinearity was checked, and model fitness was also checked using Hosmer–Lemeshow test, with a p-value > 0.05. Finally, the adjusted odds ratio (AOR) with a 95% CI was computed, and a p-value < 0.05 was used to declare statistical significance.

## Results

### Sociodemographic characteristics of the participants

A total of 415 mothers participated in the study, with a 99.5% response rate. Among those respondents, 143 (34.5%) were between age 30–34 years and their neonates postnatal age were 268 (64.6%) older than 14 days. The mean age of the mothers and the standard deviation were 30.6 (± 5.8) years, respectively and the mean age of the neonates and the standard deviation were 17.6 (± 6.9) days, respectively. Most participants, 384 (92.5%) were married, and approximately 125 (30.1%) of the mothers were unable to read and write (Table 1).

**Table 1:** Sociodemographic characteristics of the mothers with their neonates in Jigjiga City, Somali regional State, Eastern Ethiopia, 2024

Variables	Category	Frequency (*n*)	Percent (%)
Mother’s age	≤ 24 years	86	20.7
	25–29 years	71	17.1
	30–34 years	143	34.5
	≥ 35 years	115	27.7
Neonates’ age	< 15 days	147	35.4
	≥ 15 days	268	64.6
Marital status of mothers	Single	3	0.7
	Married	384	92.5
	Divorced	20	4.8
	Widowed	8	1.9
Mothers’ educational status	Can’t read and write	125	30.1
	Can read and write	107	25.8
	Primary	88	21.2
	Secondary & above	95	22.9
Mothers’ occupation	Student	18	4.3
	Housewife	276	66.5
	Gov’t employee	49	11.8
	Private employee	28	6.7
	Merchant	44	10.6
Family size	1–3	68	16.4
	4–6	221	53.3
	≥ 7	126	30.4
Husbands’ educational status	Can’t read and write	68	16.4
	Can read and write	128	30.8
	Primary	113	27,2
	Secondary & above	106	25.5

### Maternal-related factors of the participants

Most of the participants, 330 (79.5%), had ANC follow-up and about 289 (69.6%) gave birth through spontaneous vaginal delivery (SVD) (Table 2).

**Table 2:** Maternal-related factors of mothers in Jigjiga City, Somali regional State, Eastern Ethiopia, 2024

Variables	Category	Frequency (*n*)	Percent (%)
Antenatal care visit	Yes	330	79.5
	No	85	20.5
N*o* of antenatal care visits (*n* = 330)	1–3	128	38.8
	≥ 4	202	61.2
Place of delivery	Hospital	308	74.2
	Health center	75	18.1
	Home	32	7.7
Mode of delivery	SVD	289	69.6
	C/S	97	23.4
	Instrumental	29	7

### Mothers’ knowledge about neonatal sunlight exposure

Most of the mothers, 326 (78.6%), had information about the sunlight exposure of neonates. With respect to the benefit of sunlight exposure, 284 (87.1%) said that sunlight exposure had benefit for the neonates and about 268 (82.2%) mentioned that sunlight exposure had harmful effect for their neonates (Table 3).

**Table 3:** Knowledge of neonatal sunlight exposure among mothers in Jigjiga City, Somali regional State, Eastern Ethiopia, 2024

Variables	Category	Frequency (*n*)	Percent (%)
Had information about sunlight exposure	Yes	326	78.6
	No	89	21.4
Source of information (*n* = 326)	Physician	84	25.8
	Midwives/ nurses	163	50
	Television/ radio	66	20.2
	Neighbours/ elders	225	69
Is sunlight exposure beneficial? (*n* = 326)	Yes	284	87.1
	No	42	12.9
The benefit of sunlight exposure (*n* = 284)	Strengthen bone	115	40.5
	Strengthen teeth	20	7
	Keep child warm	151	53.2
	Produce vitamin D	154	54.2
	Strengthen body	175	61.6
Is sunlight exposure harmful? (*n* = 326)	Yes	268	82.2
	No	58	17.8
The harmful effect of sunlight exposure (*n* = 268)	Skin cancer	78	29.1
	Sterility	75	28
	Blindness	174	64.9
	Sun burn	39	14.6
Good time to expose neonate (*n* = 326)	Morning	266	81.6
	Mid-day	18	5.5
	Afternoon	42	12.9

### Mothers’ knowledge level about sunlight exposure of their neonates

**Fig. 1:**
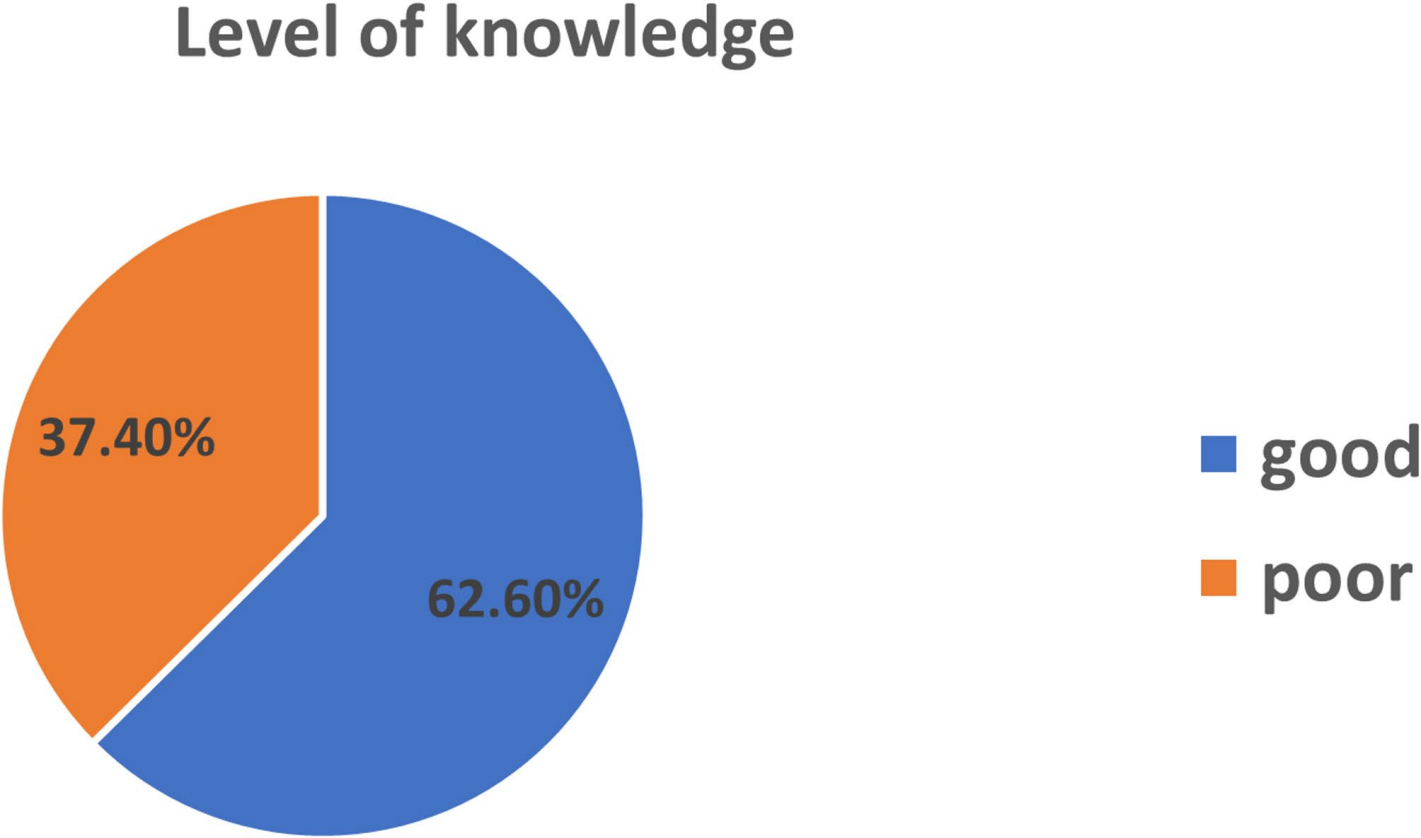
Distribution of mothers by their level of knowledge about sunlight exposure in Jigjiga city, Somali Regional State, Eastern Ethiopia, 2024

Based on the above knowledge questions, out of the total respondents, 326 (78.6%) had information about sunlight exposure and from those, about 204 (62.6%) of mothers had good knowledge based on the operational definition constituting questions such as mothers had information about sunlight, benefit of sunlight, harm of sunlight and good time to expose their neonates to sunlight (Fig. 1).

### Practice and behavioral factors of mothers with neonatal sunlight exposure

Among the 415 respondents, only 126 (30.4%) mothers exposed their neonates to sunlight. Among these, 73 (57.9%) started exposing their neonates to sunlight for ≥ 15 days. Almost half, 65 (51.6%) of the mothers exposed their neonates to sunlight at most 2 times a week. The majority, 114 (90.5%) of the mothers exposed their neonates between 8-10AM. Among the total respondents, 360 (86.7%) mothers feared exposing their neonates to sunlight (Table 4).

**Table 4:** Practice and behavioral factor of neonatal sunlight exposure among mothers in Jigjiga City, Somali regional State, Eastern Ethiopia, 2024

Variables	Category	Frequency (*n*)	Percent (%)
Do you expose your neonate to sunlight?	Yes	126	30.4
	No	289	69.6
Age of neonate to start sunlight exposure (*n* = 126)	< 15 days	53	42.1
	≥ 15 days	73	57.9
Frequency of sunlight exposure per week (*n* = 126)	< 3 days	65	51.6
	3–7 days	61	48.4
Place of sunlight exposure (*n* = 126)	Indoor	24	19
	Outdoor	102	81
Time of the day to expose neonate (*n* = 126)	Morning 8-10AM	114	90.5
	Mid-day 11-1PM	5	4
	Afternoon 2-4PM	7	5.6
Condition of clothing during sunlight exposure (*n* = 126)	Unclothed	44	34.9
	Partly clothed	68	54
	Completely clothed	14	11.1
Duration of sunlight exposure per one time(*n* = 126)	< 15 min	2	1.6
	≥ 15 min	124	98.4
Apply lubricants on neonate’s body (*n* = 126)	Yes	116	92.1
	No	10	7.9
Time of application of the lubricants (*n* = 116)	Before exposure	8	6.9
	During exposure	11	9.5
	After exposure	97	83.6
What type of lubricant is applied? (*n* = 116)	Baby Vaseline	58	50
	Baby lotion	35	30.2
	Butter	7	6
	Olive oil	16	13.8
Do you fear exposing your neonate to sunlight exposure?	Yes	360	86.7
	No	55	13.3
Mothers’ fear (*n* = 360)	Evil eye	235	65.3
	Sickness	271	75.3
	Cold	59	16.4

### Practice level of mothers with respect to sunlight exposure of their neonates

**Fig. 2:**
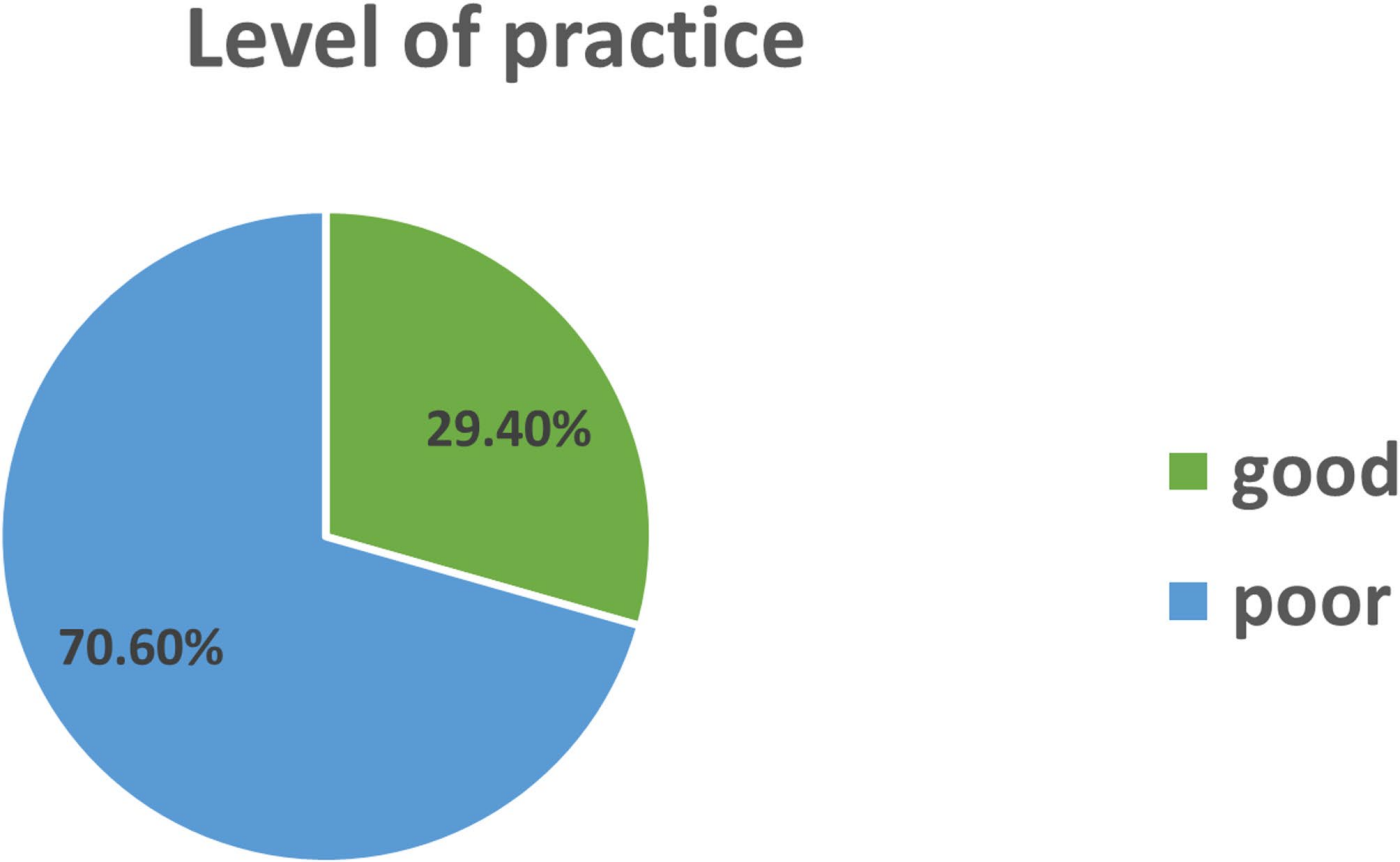
Distribution of mothers by their practice level with respect to sunlight exposure in Jigjiga city, Somali Regional State, Eastern Ethiopia, 2024

On the basis of the above practice questions, 126 (30.4%) of the mothers exposed their neonates to sunlight, and only 37 (29.4%) of the mothers had good practice (on the basis of the operational definition, respondents that answered all the practice questions correctly, such as mothers who had exposed their neonates to sunlight, the postnatal age of the neonate, the frequency, the place of exposure, the time of exposure, the condition of clothing, the duration of exposure, and when they applied lubricants) with respect to sunlight exposure for their neonates (Fig. 2).

### Factors associated with mothers’ level of practice with respect neonatal sunlight exposure

In the bivariable binary logistic regression, variables such as age and current marital status of the mother, number of ANC follow-ups, mode of delivery, knowledge level of the mother and fear of sunlight exposure were candidates for multivariable analysis at p-values < 0.25. In the multivariable binary logistic regression analysis, the current marital status of the mother, mode of delivery, knowledge level of the mother and fear of sunlight exposure were retained and showed statistically significant associations with the practice level of the mothers at p-value < 0.05, whereas the age of the mother and number of ANC follow-ups were not retained in the multivariable analysis.

Those who were married (AOR = 0.17, 95% CI: 0.03–0.92) were 83% less likely to have good practice of sunlight exposure compared with those who were divorced. Mothers who delivered through cesarean section (AOR = 0.05, 95% CI: 0.01–0.54) were 95% less likely to practice good sunlight exposure compared with those who delivered through spontaneous vaginal delivery. Mothers who had poor knowledge of sunlight exposure (AOR = 0.31, 95% CI: 0.1–0.96) were 69% less likely to have good practice than mothers who had good knowledge of neonatal sunlight exposure compared to those who had good knowledge. Mothers who did not have fear of sunlight exposure (AOR = 3.06, 95% CI: 1.02–9.17) were 3.06 times more likely to have good practice than mothers who feared exposing their neonates to sunlight (Table 5).

**Table 5:** Bivariate and multivariate binary logistic regression analysis of maternal practice level toward sunlight exposure of their neonates in Jigjiga City, Somali regional State, Eastern Ethiopia, 2024

Variables	Category	Practice level		COR (95% CI)	AOR (95% CI)
		Good	Poor		
Age of mothers	≤ 24 years	7	12	1	1
	25–29 years	7	11	1.09 (0.29–4.12)	1.76 (0.24–13.1)
	30–34 years	11	24	0.79 (0.24–2.54)	1.10 (0.17–7.02)
	≥ 35 years	12	42	0.49 (0.16–1.52)	0.93 (0.16–5.52)
Current marital status	Married	29	78	0.51 (0.19–1.4)	0.17 (0.03–0.92) *
	Divorced	8	11	1	1
Number of ANC follow up	1–3 times	8	32	1	1
	≥ 4 times	19	41	1.85 (0.72–4.78)	1.8 (0.57–5.72)
Mode of delivery	SVD	28	42	1	1
	C/S	6	27	0.33 (0.12–0.91)	0.05 (0.01–0.54) *
	Instrumental	3	20	0.23 (0.06–0.83)	0.22 (0.04–1.24)
Knowledge level	Good	27	38	1	1
	Poor	10	51	0.28 (0.12–0.64)	0.31 (0.1–0.96) *
Fear of sunlight exposure	Yes	16	55	1	1
	No	21	34	2.12 (0.98–4.62)	3.06 (1.02–9.17) *

Hosmer and Lemeshow, *p*-value = 0.085

## Discussion

In this study, the prevalence of neonatal sunlight exposure among mothers living in Jigjiga city was 29.4%, with a 95% CI (22.1–38.1). The findings of this study were consistent with those of studies conducted in Addis Ababa town (27.1%) [16] and Aleta Wondo town (32.6%) [23].

In contrast, the findings were lower than those of different studies conducted in Debre Berhan town 65.7% [17], Wolkite town (67.3%) [18], Mettu district (57.7%) [24], Yirgalem district (54.5%) [19], Farta district (54.3%) [25], Debre Markos town (44.6%) [26], Dejen district (44%) [15] and Dessie town (41.1%) [27]. The possible reason for this discrepancy might be that most of the studies used infants as their study population and most mothers start to expose their infants after 45 days, not prior to that, owing to cultural beliefs, while this study considered mothers with their neonates where the practice became low. Additionally, gaps within the local health system such as inadequate maternal education regarding the benefits of sunlight exposure, and insufficient outreach programs can hinder mothers from practicing recommended guidelines.

Another possible reason might be the cutoff point used to classify mothers’ practice level of neonatal sunlight exposure. Most of the studies used the median value as their cutoff point, as the mothers who responded correctly above the median value were categorized as those who had good practice and the rest were categorized as having poor practice. In contrast, this study classified mothers with good practice and poor practice as those who responded correctly to all the practice questions and those who did not, respectively.

Married women were less likely to practice neonatal sunlight exposure. They were found to be 83% less likely to have good practices than those who were divorced [AOR = 0.17, 95% CI: (0.03–0.92)]. This finding is consistent with a study conducted in the Mettu district [24], where divorced or widowed women had good practice compared to married women did, whereas it was in comparison with the studies conducted in Yirgalem [19], Debre Markos [26] and Addis Ababa [16] in which marital status was not significantly associated with the practice of sunlight exposure (*p* > 0.05). The possible reason why divorced women had good neonatal sunlight exposure compared to married women may be due to the higher educational level of the former, which can result in better information about sunlight exposure.

Mothers who delivered through cesarean section were 95% less likely to have good practices than mothers who delivered through spontaneous vaginal delivery were [AOR = 0.05, 95% CI: (0.01–0.54)]. This might be due to mothers not being able to expose their neonates to sunlight since they are experiencing pain and recovery time is also lightly longer.

Mothers who had poor knowledge about neonatal sunlight exposure had poorer practices than mothers who had good knowledge did; they were found to be 69% less likely to have good practice than those with good knowledge [AOR = 0.31, 95% CI: (0.1–0.96)]. This finding was consistent with studies conducted in Aleta wondo [23], Dejen [15] and Addis Ababa [16]. The reason why mothers who had poor knowledge practiced poorer might be the fact of practicing less when they do not know well.

In this study, neonates whose mothers had no fear of sunlight were 3.06 times more likely practice good neonatal sunlight exposure than were neonates whose mothers’ feared exposure to sunlight [AOR = 3.06, 95% CI: (1.02–9.17)]. This finding was consistent with studies conducted in Debre Markos [26], Farta district [25] and Addis Ababa town [16].The possible reason why mothers who had fear would have poor practice might be poor knowledge. Even though most of the mothers (62.6%) had good knowledge in this study, the source of the information might have affected their level of proper information concerning sunlight exposure as the majority had information from their neighbors/elders.

### Strengths and limitations of the study

#### Strengths of the study

Previous studies did not consider the mode of delivery, but this study investigated this factor.

Recall bias was minimized since the study emphasized only mothers with neonates.

The data were collected using Kobo, ensuring data quality.

Could be a baseline data for other researchers since there is scarcity of similar articles done in our country specifically this study area.

### Limitations of the study

The study is a cross-sectional study, which cannot establish a cause–and–effect relationship.

The study should have included qualitative data, and information from focus group discussions (FGDs) with selected mothers, health professionals, and health leaders to triangulate the quantitative data obtained from the interviews.

## Conclusion and recommendations

In this study, the prevalence of mothers who had good neonatal sunlight exposure was 29.4%, which is low compared to most of the findings of other studies. The marital status of the mother, mode of delivery, mothers’ knowledge of sunlight exposure and mothers’ fear of sunlight exposure were significantly associated with the practice level of the mothers.

Health professionals should provide health education that focuses on the importance of sunlight exposure and all the proper ways of exposing neonates to improve mothers’ practice of exposing neonates to sunlight. Finally, researchers should perform further studies to identify mothers’ practice of sunlight exposure for their neonates with qualitative data.

## Data Availability

All data about this study are contained and presented in this document.

## Abbreviations

ANC: Antenatal Care
AOR: Adjusted Odds Ratio
CI: Confidence interval
CS: Cesarean Section
SPSS: Statistical Package for Social Science
SVD: Spontaneous Vaginal Delivery
UVR: Ultraviolet Radiation
VDD: Vitamin D Deficiency
